# Motivations Influencing Alipay Users to Participate in the Ant Forest Campaign: An Empirical Study

**DOI:** 10.3390/ijerph192417034

**Published:** 2022-12-18

**Authors:** Shujie Wang, Mohammed Habes Ibrahiem, Mengyu Li

**Affiliations:** 1 Department of Media & Communication, Kangwon National University, Chuncheon-si 24341, Gangwon-do, Republic of Korea; 2 Radio & Television Department, Faculty of Mass Communication, Yarmouk University, Irbid 21163, Jordan; 3 College of Humanities and Development Studies, China Agricultural University, Beijing 100083, China; 4 Faculty of Modern Languages and Communication, Universiti Putra Malaysia, Serdang 43400, Selangor, Malaysia; 5 School of Journalism and Communication, Zhengzhou University, Zhengzhou 450001, China

**Keywords:** motivation theory, uses and gratifications theory, satisfaction, Alipay, Ant Forest, China

## Abstract

As one of the largest payment platforms in China, Alipay, launched a green behavior project called Ant Forest. The purpose of taking this initiative by Alipay is to make the world greener. This mobile application has motivated many users to plant millions of trees. However, minimal studies have been conducted to empirically disclose the factors that motivate Alipay users to participate in Ant Forest. A mixed-method approach is used to examine the factors influencing Alipay users to participate in Ant Forest and the level of satisfaction they got from it. The qualitative method was carried out through a structured questionnaire from 400 Alipay users and qualitative data from 20 users who have applied to Ant Forest and successfully planted trees. Additionally, alongside the mixed method employed to rationalize the relationship highlighted, a multiple regression model was employed to predict the value of a dependent variable (level of satisfaction) based on the value of several independent variables (namely, number of years engaged with Ant Forest activities, age, gender, profession, and education). The empirical results show that intrinsic motivations (enjoyment, social interaction, fulfilment, altruism) and extrinsic motivations (external rewards, competition) influence Alipay users to participate in the Ant Forest project. Further insights are presented in the concluding section for all stakeholders for environmental sustainability among the users of Alipay.

## 1. Introduction

The prevention of global environmental degradation is of utmost importance in today’s world. The rapid increase in global warming and climate change results from how rapidly the environment is deteriorating [1]. Environmental degradation is mainly caused by anthropogenic activities that border around industrialization, urbanization, desertification, population increase, deforestation, and an increase in the energy consumption of natural resources [2]. The degradation of the environment not only impacts the climate but also has a major impact on the human and animal populations [3,4]. It leads to increased climatic, natural disasters and a depletion of the ozone layer, which brings harmful radiation to our planet, causing incurable diseases such as cancer [5].

One of the key precursors to desertification in China is deforestation [6,7]. The growing domestic needs are the leading cause behind China’s environmental desertification and burning forests, etc. [8]. Overgrazing and farming practices make the soil lose its natural texture and vitality. Incessant fossil fuel usage has led to unequal rainfall distribution, bringing about Chinese climate change (i.e., triggering desertification) [9]. Moreover, rising temperatures in some parts of China take away soil moisture and dry up surface water bodies [10,11]. The economic importance of forests and the subsequent worldwide wood trade makes them vulnerable to logging [12]. Chinese environmental desertification affects many people, particularly the poor in drylands [13]. It reduces soil fertility and organic matter content. Wind and water also lead to accelerated soil erosion [14]. In such a situation, Chinese people need to campaign for more gardens, green parks, and planting trees everywhere, and green carbon finance is put forward and practiced [15].

**Figure 1:**
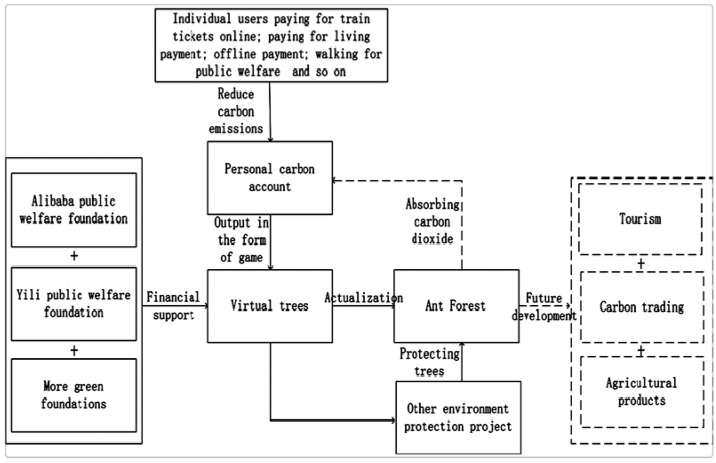
The development model of FinTech Alipay from the perspective of Ant Forest.

Over the past years, China and the international community have been working on sustainable finance, especially climate finance China Council for International Cooperation on Environment and Development Secretariat 2022 [16]. Strengthening ecological and biodiversity protection is an important measure to maintain China’s ecological security and improve people’s well-being and it is a critical practice to create social welfare. In addition to passive measures (e.g., taxation to stop activities that damage the environment), it can actively promote environmental protection concepts to the public and hopefully change people’s behavior. FinTech (financial technology) plays an important role here, where promotion can be achieved in innovative ways. An example is Ant Forest, a gamified green initiative launched by Alipay, the biggest FinTech Company in China, of which the aim was to combat climate change [17]. Established in 2004, Alipay (China) Network Technology Co., Ltd. (Hangzhou, China). is a third-party payment platform in China dedicated to providing “simple, safe, fast and convenient” payment solutions for enterprises and individuals. As a featured project of Alipay Corporation, Ant Forest aims to cultivate its mobile app users’ green consumption habits. The Ant Forest campaign encourages users to choose low-carbon and environmentally friendly consumption methods. App users may trade their virtual energy points, which accumulate for low-carbon behaviors, for the opportunity to plant trees in desertified lands. This green campaign invites the public to participate in collective action to beautify the earth so that individuals can contribute to environmental protection and public welfare activities [11]. The low-carbon activities include commuting with public transportation, walking, and online ticket purchase, etc. Users can interact with each other by “stealing” or sharing green energies from their app friends [18]. The user base of Ant Forest is large and growing rapidly. After its launch in 2016, more than 230 million users have planted 10.25 million trees in the Inner Mongolia region of China, reducing carbon emissions by 1.22 million tons [19]. The users of Ant Forest cover different age groups from the 1970s to the post-2000s. If we have a customer group with these different age groups, we have different psychological characters and needs. Therefore, Ant Forest chose a business model that brings the values of the satisfaction of the public, psychological satisfaction, helping the planet, and social entertainment [20]. The proposed Ant Forest business model and its future development in combining the “Internet + Green Finance” [21] is shown in Figure 1.

This paper first focuses on the research literature on uses and gratifications theory and motivation theory, followed by an explanation of the critical concepts regarding the relationship between usage motivation and satisfaction with Ant Forest among users. The scholars Tamborini et al. [22], Reinecke et al. [23], Wang et al. [24], Siyal et al. [25], and Choi [26] took into account the relationship between media usage motivation and satisfaction, thus helping to explain and discuss the theoretical importance of the Ant Forest project. The potential and actual conflicts between online public welfare projects and user participation have drawn the attention of researchers [27,28,29]. However, such conflicts are rarely articulated in theoretical terms. Many papers deal with online public interest projects from the perspective of green purchase attitudes and green purchase intentions based on the theory of the planned behavior model [30], as a platform to improve firm performance and environmental corporate social responsibility [21,31,32], or from the perspective of Being Green with Fintech to explore the environmental value of Ant Forest and the real impact it has on the participants’ consumption behavior [17].

The motivation theory could be applied to satisfy intrinsic and extrinsic needs [33]. The incentive theory suggests that individuals are motivated if they are rewarded. Individuals derive motivation from internal and external rewards [34]. Intrinsic motivation exhibits self-rewarding behavior, which drives a person to indulge in certain tasks because of self-seeking pleasure [35]. These factors include enjoyment [36,37] social interaction [38], fulfillment [39], and altruism [40,41]. On the other hand, extrinsic motivation refers to behavior resulting from external factors or baits such as rewards. This type of motivation arises from outside the individual, as opposed to intrinsic motivation, which originates inside the individual. Extrinsic motivation contains factors of external rewards and competition [42].

The uses and gratifications theory (UGT) show how an individual uses media for purposes such as relaxation, entertainment, and socializing [43]. Mainly, people seek three types of gratification from media: emotional, wishful thinking, and learning [44]. Daily soaps are meant to provide the first two emotional needs: emotions, such as to watch somebody being loved or loving, revenge, anger, etc., and wishful thinking, such as dreams becoming true or everything becoming positive. Therefore, this theory explains how users look for media to fulfil their different needs, such as information, social interactions, gaining knowledge, or just feeling good about themselves [45]. The research based on the UGT has also revealed that, among the gratifications on social media, young people in particular are meeting new people, satisfying teenagers’ need to belong and be entertained [46].

Though Ant Forest came with great success, limited research has helped FinTech policymakers with the sustainable growth of the app users’ green behavior. In addition, since no study has been performed to empirically disclose the factors that motivate Alipay users to participate in Ant Forest, the primary purpose of doing this study is: (1) to discover the motivation factors that influence Alipay users to join Ant Forest and (2) to predict the value of the “level of satisfaction” based on the value of “number of years engaged with Ant Forest Activities, age, gender, profession, and education”. The research questions for the quantitative part are: (1) Do intrinsic motivations and extrinsic motivations influence Alipay users to participate in the Ant Forest project? (2) If yes, what motivations affect Alipay users to participate in Ant Forest? (3) What is the relationship between the level of satisfaction and the number of years engaged with Ant Forest activities, age, gender, profession, and education? Moreover, the research questions for the qualitative part are: (1) What are the fun factors in Ant Forest? (2) What is the purpose of participating in Ant Forest? (3) Why are participants interested in participating in such an initiative that protects the environment? (4) What type of interaction do they have with other friends in Ant Forest? (5) Do they get any appreciation from anywhere because of Ant Forest? (6) Where and how do respondents find the energy to participate in Ant Forest?

Ant Forest is a comparatively new and innovative field of Alipay which has yet to be studied extensively and from the perspectives of the UGT and the theory of intrinsic and extrinsic motivation. New frameworks have been incorporated to interpret the Ant Forest phenomenon in this study. With the goal of better understanding the interaction between online public welfare projects and the participants, this study could help Alipay improve its user satisfaction. Other policymakers might also understand the motivations that influence Chinese people to participate in online environmental protection and public welfare projects.

Although projects led by green finance and public welfare environmental protection have the potential to contribute to China’s environmental protection and economic development [47], less attention has been paid to the motivation and satisfaction of existing active users of Ant Forest. This paper explores this issue by combining the theory of uses and gratifications and the motivation theory.

The research framework of this study extends the uses and theoretical gratifications model, focusing on the usage motivation and satisfaction of Ant Forest users and the variables of motivation theory in terms of motivation factors. In this study, demographic factors are also considered control variables to discuss the impact of users’ satisfaction. Ant Forest is an online public welfare project, and users participate in it to achieve the purpose of environmental protection and increase their experience value.

## 2. Methods

### 2.1. Mixed Methods

We used qualitative and quantitative methods, procedures, theories, and concepts to conclude the motivational factors influencing Alipay users to participate in the Ant Forest project. This allows us to compensate for the weakness of using only one research design method and capitalizing on the strengths of using either qualitative or quantitative methods [48]. The mixed method is sequential in this research [49]; therefore, priority is given to the respondents’ background information and the quantitative set of questions [50]. Quantitative questions are asked first, and the next set is qualitative [51]. In this, the data evaluation is first done for the quantitative questions, then qualitative, and then these two are linked.

Moreover, two separate data strands are made: one for quantitative and one for qualitative [52]. The interviews and questionnaire results are integrated with the number to provide better insight. The mixed method provides accurate measures and quality.

### 2.2. Research Design for the Quantitative Study

Demographic factors affect continuance intention and behavior [53]. For example, among drivers who have different jobs, the continuous use of dynamic transport-sharing services is considered a pro-environmental and pro-social behavior [54]. Williams-Nwagwu et al. [55] found that the user’s age differentiates satisfaction with media technology. Then, other scholars found that individuals with higher educational qualifications tend to be more obsessed with using communication technologies for various purposes [56]. Moreover, in previous research on Ant Forest, demographic information such as age, gender, income, and highest education level are included as control variables [57]. Here, in this study, the relationship between the level of satisfaction and the number of years engaged with Ant Forest activities, age, gender, profession, and education are discussed. For example, the demographic profession information is measured by salary, working years, and hierarchy.

The population of this study is the people who use the Ant Forest project. The total number of users of the Ant Forest is over 200 million. A set of questionnaires has been designed to collect the quantitative data. This questionnaire involves specific yes–no and multiple-choice questions provided to the selected population group. The questionnaire of this study was adopted and adapted from the questionnaires of previous relevant studies. The measurement items of the dependent variable (satisfaction) were adapted from Chyi et al. [58], Reinecke et al. [59], Reinecke et al. [23], Kim et al. [60], Chiou et al. [61], Liu et al. [62], and Liu et al. [63]. For the independent variables, the measurement items were adapted from Huang and Hsu [64], Bellman et al. [65], Liao et al. [66], Fırat et al. [67], and Carvache-Franco et al. [68]. This questionnaire is mainly designed to understand the feelings or opinions regarding the motivational factors influencing them to participate in Alipay’s Ant Forest project. To obtain the sample, we first compiled a list of 1000 people who have an Alipay account and Internet access. To collect the numerical data, questionnaires were distributed using the online platform powered by Wen Jun Xing (问卷星 in Chinese). Links to the questionnaire were then sent by WeChat, QQ, and Weibo (China’s top three largest social networking apps). Then, four hundred respondents were randomly selected who had also opened Ant Forest and planted trees successfully. The quantitative data were then analyzed using SPSS 19.0 software. Descriptive statistics and a multiple linear regression model were used to interpret the data.

### 2.3. Research Design for the Qualitative Study

For the qualitative part, the sample size is twenty. Respondents expressed their views and opinions about the motivation that influenced them to participate in Alipay’s online environmental protection and public welfare project. The semi-structured interview was conducted with the participants to collect non-numerical data, which provided qualitative data to compare with the previous information [69]. Unlike following any strict formal questions, it asks more open-ended questions to obtain responses about the study. A semi-structured interview is appropriate for this study, as it is open and flexible and gives us more opportunity to talk to the respondents to gather the right information about the motivations that influence them to participate in the online environmental protection and public welfare project of Alipay. The questions from the questionnaire are shown in Section 3.2. Content analysis is used to analyze the qualitative data collected from 20 respondents.

### 2.4. Ethics Considerations

This research involving human participants was reviewed and approved by the Ethics Committee of Zhengzhou University, PR China (Issue date: 24 August 2020). The respondents provided their consent with electronic or written signatures to participate in this study. All respondents know that all the personal information related to this study will be kept confidential and used only for the research purposes of this paper. They have been informed that no personal information or data will be shared with any third party.

## 3. Results

### 3.1. Quantitative Study

#### 3.1.1. Demography of the Quantitative Study

Detailed demographic information is provided in Table 1. Among the 400 respondents, the highest education level reached by 5 is primary school or below, by 27 is high school, by 290 is a bachelors, by 49 is a masters, and by 29 is a PhD and above. In this study, 73% of respondents were students, 14% were job holders, 8% were businessmen, and 6% were from other professions.

#### 3.1.2. Correlation and Multiple Regression Model

This section first uses correlation analysis to test the correlation significance between independent and dependent variables. The post hoc regression analysis was further performed if there was a verified significant correlation. The multiple regression analysis could then clearly show whether the independent factor has a strong and positive influence on the dependent variable. Moreover, a mediating or moderating effect was not involved in this present research. For the nominal variable (e.g., gender), ordinal values were assigned to make the Pearson’s R analysis between independent and dependent variables more convenient (e.g., male = 1, female = 2).

The Pearson correlation matrix between factors and the level of satisfaction is presented in Table 2. The dependent variable is the level of satisfaction towards the Ant Forest from the uses and gratifications theory. Level of satisfaction correlated significantly with the number of years engaged with Ant Forest (R^2^ = 0.644) and education level (R^2^ = −0.1111) at the 0.01 and 0.05 levels (two-tailed), respectively. It is found that the value of R is 0.649 (Table 3), which indicates 65% of the dependent variable (the level of satisfaction) is verified by the independent variables (including number of years engaged with the Ant Forest Activities, gender, age, profession, and education). In addition, the value of R squared is 0.421, which means the independent variables explain 42% of the dependent variables. It is seen that sum of the squares of regression is 161.316. Moreover, the mean square and F test results are 32.263 and 57.383, respectively.

From Table 4, the coefficient for gender is 0.047, which indicates that gender is positively related to the level of satisfaction from the Ant Forest project. Age is also positively related to the level of satisfaction, as the coefficient of age is 0.034, which is positive. Education and profession are negatively related to the level of satisfaction from the Ant Forest project. However, the correlation is not statistically significant (i.e., *p*-value > 0.05). Finally, the number of years engaged with Ant Forest Activities is positively related to the level of satisfaction (*p*-value < 0.001). If the year of engagement is increased by one unit, then the satisfaction level is increased by 0.623. It can be said that a person with more years of engagement in the Ant Forest project is positively related to a higher satisfaction in Alipay’s environmental protection activities. It means older members of Ant Forest might gain higher satisfaction from Alipay’s environment protection activities compared to new members of Ant Forest. As the value of R squared is 0.421 (Table 3), 42% of the satisfaction level from Ant Forest project activities is explained by the number of years engaged with Ant Forest Activities, gender, age, profession, and education. The results, in general, are statistically significant.

After constructing the multivariate regression model, we found its results fit the standard model. According to the model summary, the value of R squared is 0.421 (Table 3), indicating that 42% of the satisfaction level from Ant Forest project activities is explained by the number of years engaged with Ant Forest Activities, gender, age, profession, and education. The results, in general, are statistically significant. Table 4 then shows the standard coefficient and the statistical significance between independent and dependent variables (i.e., level of satisfaction). The number of years engaged with Ant Forest Activities is positively related to the level of satisfaction, and their correlation is significant (*p*-value < 0.001). If the year of engagement is increased by one unit, then the satisfaction level is increased by 0.623. It can be said that a person with more years of engagement in the Ant Forest project is positively related to higher satisfaction in Alipay’s environment protection activities, which means older members of Ant Forest might gain higher satisfaction from Alipay’s environment protection activities compared to the newer members of Ant Forest. For other independent variables, the coefficient for gender is 0.047, which indicates that gender is positively related to the level of satisfaction from the Ant Forest project. Age is also positively associated with satisfaction, as the age coefficient is 0.034. Education and profession are negatively related to the level of satisfaction from the Ant Forest project. However, the correlation is not statistically significant (i.e., *p*-value > 0.05).

#### 3.1.3. Descriptive Statistics of the Survey Results

In Table 5, by evaluating the intrinsic motivations, 83.8% of respondents said that the way Ant Forest is played is enjoyable (tension-free needs). In addition, only 16.3% did not find any enjoyment in the Ant Forest. Furthermore, 90% of respondents said that protecting the environment is vital. It is seen that 89% of respondents said that environmental degradation is a very concerning issue for them. Moreover, 64.5% of respondents think social interaction played a very important role in participating in Ant Forest. However, 35.5% said that they are not motivated by the social network, cooperation, and community, but rather by other issues. Most of the respondents are motivated by external rewards. It is observed that 96.5% of users said that they joined Ant Forest to contribute to society based on positive affective needs. Furthermore, 55% of users were motivated by competition among their friends, and 45% were not motivated by the competition.

### 3.2. Qualitative Study

#### 3.2.1. Intrinsic Motivations

*Enjoyment and tension-free needs: What are the fun factors in the Ant Forest?*

Among the 20 respondents, 16 expressed that the enjoyment is found in “virtually monitoring the growth of trees and optimize the environmental effect of reality in real-time”. Another four respondents said, “I can also get the green energy from their friends’ accounts, so I think it is also very fun”. The respondents’ perception of enjoyment of joining Ant Forest is understood by asking this question.

Fulfilment: What is the purpose of participating in Ant Forest?

They all stated, “I think protecting the environment is a very vital issue for us”. Most respondents (18 out of 20) said, “the purpose of my participating in the Ant Forest is to contribute to environment protection”. Of the respondents, 12 said, “Ant Forest encourages me to practice low-carbon behaviors”. Of the respondents, 8 said that ensuring social welfare is very important for them. This question helps to understand the elements that could help them to build fulfilment.

*Altruism and cognitive needs: What are the reasons for interest in participating in such initiatives that protect the environment?*

All the participants said, “I think environmental degradation is a very concerning issue for us”. Among 20 respondents, 15 believe that “Ant Forest helps Chinese people to improve their awareness of environmental protection and form a good atmosphere”. In the answers, respondents expressed feelings of “caring for environment and public well-being” and considered themselves “self-determined and self-conscious” about engaging in this green initiative.

Social interaction and affective needs: What types of interactions do respondents have with other friends in Ant Forest?

It was found that among 20 respondents, 12 were motivated by social networks, cooperation, and community. Therefore, the purpose is similar to social media, which boosts their motivation. Of the respondents, 14 said that “they can accumulate energy to exchange for opportunities to plant trees, and invites their friends to participate in the collective action to beautify the earth”.

#### 3.2.2. Extrinsic Motivations

*External rewards: Do respondents get any appreciation from anywhere because of Ant Forest?*

Owning a real-life tree could motivate them to participate in Ant Forest. Most respondents believe they insist on practicing low-carbon environmental protection behavior for public welfare. In this way, they obtain a green forest for wind and sand protection, which is undoubtedly of great practical significance for optimizing the living environment for the public and improving the air quality around the world. In this way, they can gain a sense of honor and a sense of achievement for public welfare.

*Competition: Where do they find the energy to participate in Ant Forest?*

The semi-structured interview found that the competition mechanism motivates them to pay more attention to Ant Forest. Of the respondents, 13 said that “by increasing green energy, I can share it with other friends by virtually watering their trees”. Some others also take the initiative to increase their green points. Through this mode of competition, they can be motivated to participate actively in Ant Forest.

## 4. Discussions

In the era of Internet public welfare, green finance and public welfare projects have now been transferred online and have become quite diversified [70]. The full integration of the Internet and charity thus inspired creativity in the industry, paving the way for organizational transformation and innovation [71]. The public welfare projects have been upgraded from the old passive purchase donation method to a new method of voluntary donation after purchase [72]. The mobile application of Ant Forest can somehow leverage consumer behavior or green financial behavior more broadly and proactively for public welfare, thereby motivating the public to participate in environmental protection [18]. 

Drawing on the UGT, we examined the motivational factors (intrinsic and extrinsic) for the Ant Forest campaign among app users. First, consistent with the literature [73], we verified again that the continuous usage of Ant Forest (number of years engaged with Ant Forest activities) positively related to the level of satisfaction (Table 2). Though we found a negative relation between the highest education level (i.e., one of the demographic factors) and satisfaction, the *p*-value of the education coefficient is above 0.05, which indicates false positive data.

Regarding the motivational factors, the results show that intrinsic and extrinsic motivations influence Ant Forest users to engage in this green activity. Intrinsic motivations such as enjoyment and tension-free needs, fulfilment, altruism, and cognitive needs, and social interaction and affective needs, and extrinsic motivations such as external rewards and competition strongly influence the respondents to participate in Alipay’s online environmental protection activities and public welfare projects. Previous research supports that rewards and benefits ultimately lead to the motivation level for or engagement in a certain task or activity [74]. By evaluating the quantitative results, 90% of respondents admit that playing Ant Forest is pleasurable. Among them, the majority believed external rewards played very important roles. Our research demonstrates that the reward (e.g., trading virtual green energy for a real tree planted somewhere) for activities (i.e., choosing low-carbon and environmentally friendly consumption methods), as an external motivational factor, is also a favorable predictor toward Ant Forest’s continuing engagement. Researchers also believe that individuals with higher levels of bonding, care, and concern toward society and the environment intend to use products or services that reduce negative environmental impacts [75]. It is found that altruism plays an important role in driving the Ant Forest continuation intention. Of the respondents, 90% said that protecting the environment is vital for them, motivating them to contribute to social welfare activities. In addition, for 64.5% of respondents, social interaction played a very important role in participating in Ant Forest. This suggests that, by actively stimulating Ant Forest users’ motivation, environmentally conscious users may provide positive feedback. Interestingly, there were significant results in the effects of internal and external factors such as usage motivation and satisfaction. However, from the qualitative results, there seem to be some differences between the two, but they are not obvious. Therefore, usage motivation can directly affect users’ satisfaction, but this paper does not discuss the mediating effect of some variables.

Due to the game’s characteristics and the fact that users only need short bursts of time to play, Ant Forest has done a great job in influencing Alipay users to participate in public welfare projects. The positive behavior of users can be transformed into charitable donations and interests through the game, bringing more fun to users. In addition, users can also steal “energy” from other users, and a ranking show who among the user’s friends donated more “green energy”. This social interaction function also increases users’ adherence to charity, which benefits the establishment of Alipay’s social attributes.

The rapid development of the Chinese economy requires the public to adapt to a high-efficiency and fast-paced lifestyle [76]. Alipay Ant Forest uses sensor technology to monitor how users travel, which motivates users to choose low-carbon and environment-friendly walking and cycling transport to obtain green energy [77]. From the results, it is found that, through the practice of low-carbon behavior, users not only enhance their physical fitness but also their awareness of environmental protection at the same time, to achieve the implementation of the low-carbon concept. The respondents who shoulder the responsibility of environmental protection have realized the importance of protecting the environment. Driven by the appeal of environmental protection [57], the public choose the public welfare financial product Alipay Ant Forest, which has become an inevitable trend of the times.

Compared with other factors, satisfaction was more affected by tension-free needs than other independent variables. This suggests that Ant Forest’s two main game design strategies—the concept of environmental protection and the popularization of green carbon finance knowledge—do not seem to have a real effect, that is to say, to generate positive environmental engagement among users to demonstrate their motivation and satisfaction to participate in online public welfare projects [17,78]. In addition, among Ant Forest users, the qualitative results show that some users realize the importance of environmental protection and the sense of honor brought by Ant Forest, and, due to the expression of their value, are more motivated to use it, which is consistent with the findings of some previous studies [79,80]. The results show that the motivation of Ant Forest users had a significant positive impact on their satisfaction. This suggests that the satisfaction and enjoyment derived from the usage of Ant Forest can strengthen the link between users’ motivation and their sense of responsibility for environmental protection, reflecting the existing literature [25,81].

Protecting the environment is an eternal theme of our times. Setting up and putting the idea into practice that clear water and green mountains are mountains of gold and silver was written in the report of the 19th National Congress of the Communist Party of China [57]. Environmental protection is a concern that plays a vital role in our daily life. However, the weak awareness of environmental protection among contemporary people makes it difficult to carry out environmental protection behaviors. Therefore, the Alipay Ant Forest program can improve the environmental protection awareness of the Chinese people. Institutions are also directed to social media and work to improve their corporate reputation in these environments [82].

## 5. Conclusions

The primary purpose of the Ant Forest project is to convert energy into real trees. It is strongly innovative and profoundly significant. Compared with actual tree planting behavior, accumulating green energy in the Alipay Ant forest requires users to make a persistent effort. The trivial daily behavior practice of the low-carbon concept accumulates over a long period from a quantitative change to a qualitative change. The ants pay treasure forest trees to green energy converted into reality by selecting the tree species, planting place, and time to plant trees. This paper found that old members of Ant Forest gain higher satisfaction from Alipay’s environment protection activities compared to the new members. We interpret the contribution of Alipay’s Ant Forest based on the UGT model. It adds value to its customer by offering public welfare, helping the planet, psychological satisfaction, and social entertainment. The results show that Ant Forest users were motivated by social networks, cooperation, and community. It is further found that external rewards, such as competition among friends, played very important roles. It can be concluded that intrinsic and extrinsic motivations influence Alipay users to participate in Ant Forest. Specifically, enjoyment, fulfilment, altruism, social interaction, external rewards, and competition play a significant role in these online environmental protection and public welfare activities.

This study examined the relationship between Alipay users’ motivation and satisfaction with Ant Forest. The results indicate a positive and significant relationship; therefore, it is necessary to encourage the development of green carbon finance and online environmental protection projects in mainland China. This was done by testing Ant Forest users’ motivations and attitudes toward environmental protection. In this case, the outcomes show specific intrinsic and extrinsic motivation drivers in their causal chain relationships of satisfaction. Accordingly, the results indicate that the motivation of Ant Forest users plays a positive role in their satisfaction with the service. The sense of achievement with environmental awareness and virtual incentives (planted real trees in desert areas) is the true driver of Ant Forest usage and satisfaction. Most Ant Forest users use it for internal motivations such as environmental awareness, enjoyment, and a sense of accomplishment. Such motivations increase the level of satisfaction directly and indirectly. When Ant Forest helps the users plant a tree and issues a certificate, the sense of achievement and the interaction and competition with their friends can provide their satisfaction with the game, and vice versa makes the relationship between usage and satisfaction stronger.

Although Ant Forest still presents insufficient skill and experience, it can be concluded that the current situation has aroused users’ enthusiasm for environmental protection and increased their engagement with Alipay apps. From the perspective of practical application, this study provided a certain reference for the developer of Ant Forest to improve it. In addition, it also encouraged more enterprises, such as Alipay (Alibaba Group), to organize and launch online public welfare projects covering environmental protection, medical treatment, and disability, which would help enterprises gain more social prestige and reputation among consumers. More importantly, this environmental protection project has been implemented in northwest China and has achieved fruitful results. It has grown trees and enhanced Chinese people’s understanding and knowledge of green carbon finance. It has also called on more people to use Ant Forest, contributing substantially to China’s environmental protection.

The limitation of this study is that the quantitative and qualitative data have been collected from only 400 and 20 respondents, respectively. This limited number of respondents may not represent the more than 500 million participants in Ant Forest (i.e., we found that 73% of the respondents in the quantitative research are students). Another limitation is that of the data were collected online. It cannot be said that all the respondents provided absolute answers. These research results only represent our selected intrinsic and extrinsic motivation factors, so there may be other motivations that also influence them to participate in Ant Forest. Therefore, it is recommended that, in future studies, it may be possible to obtain better results by increasing the sample size and conducting a physical interview/survey.

## Figures and Tables

**Table 1:** Sampling description and distribution of Ant Forest users’ profiles.

Profile	Frequency	Percentage (%)	Valid Percent (%)	Cumulative Percent (%)
Gender				
Male	260	65.0	65.0	65.0
Female	140	35.0	35.0	100.0
Age				
Below 20 years	105	26.3	26.3	26.3
20 to 30 years	97	24.3	24.3	50.5
31 to 40 years	76	19.0	19.0	69.5
41 to 50 years	65	16.3	16.3	85.8
Over 50 years	57	14.2	14.2	100.0
Highest education				
Primary School	5	1.3	1.3	1.3
High School	27	6.8	6.8	8.0
Bachelors	290	72.5	72.5	80.5
Masters	49	12.3	12.3	92.8
PhD and above	29	7.2	7.2	100.0
Profession				
Student	290	72.5	72.5	72.5
Job Holder	55	13.8	13.8	86.3
Business	32	8.0	8.0	94.3
Others	23	5.8	5.8	100.0

**Table 2:** Pearson correlation matrix of the quantitative study.

	Gender	Age	Education	Profession	Number of Years Engaged with Ant Forest Activities	Level of Satisfaction
Gender	1					
Age	0.091	1				
Education	0.082	0.554 **	1			
Profession	0.097	0.531 **	0.455 **	1		
Number of years engaged with Ant Forest Activities	0.007	−0.007	−0.073	−0.047	1	
Level of satisfaction	0.035	−0.012	−0.111 *	−0.061	0.644 **	1

Note: **. Correlation is significant at the 0.01 level (two-tailed); *. Correlation is significant at the 0.05 level (two-tailed).

**Table 3:** Model summary and ANOVA of the quantitative study.

ANOVA ^a^						
Model		Sum of Squares	*df*	Mean Square	F	*p*-Value
1 ^b^	Regression	161.316	5	32.263	57.383	0.000 ^c^
	Residual	221.524	394	0.562		
	Total	382.840	399			

Note: ^a^. Dependent Variable: Level of satisfaction; ^b^. R = 0.649, Adjusted R^2^ = 0.414, Std. error of the estimate = 0.750; ^c^. Predictors: (Constant), Number of years engaged with Ant Forest Activities, Gender, Age, Profession, and Education.

**Table 4:** Coefficients of the quantitative study.

Coefficients ^a^						
Model		Unstandardized Coefficients		Standardized Coefficients	t	*p*-Value
		B	Std. Error	Beta		
1	(Constant)	0.626	0.130		4.825	0.000
	Gender	0.047	0.052	0.035	0.907	0.365
	Age	0.034	0.036	0.047	0.941	0.347
	Education	−0.088	0.050	−0.083	−1.757	0.080
	Profession	−0.023	0.050	−0.021	−0.460	0.646
	Number of years engaged with Ant Forest Activities	0.623	0.038	0.637	16.555	0.000

Note: ^a^. Dependent Variable: Level of satisfaction.

**Table 5:** Descriptive statistics of the survey results in general.

Items		Frequency	Percent (%)	Valid Percent (%)	Cumulative Percent (%)
Enjoyment of Ant Forest					
Valid	Yes	335	83.8	83.8	83.8
	No	65	16.3	16.3	100.0
	Total	400	100.0	100.0	
Protecting environment is a vital issue for participating in Ant Forest					
Valid	Yes	360	90.0	90.0	90.0
	No	40	10.0	10.0	100.0
	Total	400	100.0	100.0	
Awareness of environmental degradation is important					
Valid	Yes	356	89.0	89.0	89.0
	No	44	11.0	11.0	100.0
	Total	400	100.0	100.0	
Users motivated by the social network, cooperation, and community					
Valid	Yes	258	64.5	64.5	64.5
	No	142	35.5	35.5	100.0
	Total	400	100.0	100.0	
Participating in Ant Forest is a contribution to society					
Valid	Yes	386	96.5	96.5	96.5
	No	14	3.5	3.5	100.0
	Total	400	100.0	100.0	
Competition of using Ant Forest					
Valid	Yes	220	55.0	55.0	55.0
	No	180	45.0	45.0	100.0
	Total	400	100.0	100.0	

## Data Availability

Everyone who concerns about this topic and can obtain the data generated during the study from shu-jiewang@kangwon.ac.kr.
